# SERS performance of GaN/Ag substrates fabricated by Ag coating of GaN platforms

**DOI:** 10.3762/bjnano.14.46

**Published:** 2023-05-03

**Authors:** Magdalena A Zając, Bogusław Budner, Malwina Liszewska, Bartosz Bartosewicz, Łukasz Gutowski, Jan L Weyher, Bartłomiej J Jankiewicz

**Affiliations:** 1 Institute of High-Pressure Physics, Polish Academy of Sciences, Sokołowska 29/37, 01-142 Warsaw, Poland; 2 Faculty of Advanced Technologies and Chemistry, Military University of Technology, gen. Sylwestra Kaliskiego 2, 00-908 Warsaw, Poland; 3 Institute of Optoelectronics, Military University of Technology, gen. Sylwestra Kaliskiego 2, 00-908 Warsaw, Poland

**Keywords:** GaN/Ag, magnetron sputtering, nanofabrication, pulsed laser deposition, SERS substrates, surface-enhanced Raman spectroscopy (SERS)

## Abstract

The results of comparative studies on the fabrication and characterization of GaN/Ag substrates using pulsed laser deposition (PLD) and magnetron sputtering (MS) and their evaluation as potential substrates for surface-enhanced Raman spectroscopy (SERS) are reported. Ag layers of comparable thicknesses were deposited using PLD and MS on nanostructured GaN platforms. All fabricated SERS substrates were examined regarding their optical properties using UV–vis spectroscopy and regarding their morphology using scanning electron microscopy. SERS properties of the fabricated GaN/Ag substrates were evaluated by measuring SERS spectra of 4-mercaptobenzoic acid molecules adsorbed on them. For all PLD-made GaN/Ag substrates, the estimated enhancement factors were higher than for MS-made substrates with a comparable thickness of the Ag layer. In the best case, the PLD-made GaN/Ag substrate exhibited an approximately 4.4 times higher enhancement factor than the best MS-made substrate.

## Introduction

Surface-enhanced Raman spectroscopy (SERS) is a highly sensitive and specific technique with multiplexing capabilities [1–4]. It is considered for use in various applications, including biosensing and bioanalysis [4–7], and many defense and homeland security applications [8–10], such as forensic science [11] or detection of hazardous materials [12–16]. Any SERS technique application requires efficient, reproducible, and reliable SERS substrates, which often must be tailored toward specific applications [15,17–18]. The SERS substrates described in the literature include nanoparticles, core–shell nanoparticles, semicontinuous metal films, and many other nanostructures most commonly made of gold or silver [18–22]. Due to the easiness of fabrication, the most extensively studied SERS substrates are those based on metallic nanoparticles and their assemblies [21–22].

Among SERS substrates fabricated using physical methods of particular interest are those based on nanostructured GaN platforms coated with plasmonic metals or their alloys [23–32]. The chemical and electrochemical etching of GaN heteroepitaxial layers leads to various nanostructures formed on line defects (dislocations), such as straight nanopillars, bunches of nanopillars, and pits [31–32]. The nanostructured GaN platforms can then be coated with Au [26,28], Ag [25], AuCu, or AuAg alloys [24,29–32] to produce highly reproducible and efficient SERS substrates. GaN-based SERS substrates have been used so far for trace detection of explosive materials [33], fentanyl and bacterial spores’ analysis [31], gene mutation identification [34–35], and investigations of the reactivity of organic monoradicals [36].

In our previous studies, nanostructured GaN platforms were coated with pure metals or alloys using magnetron sputtering (MS) in an argon atmosphere [28–32]. Until now, no other physical vapor deposition (PVD) methods have been tested to replace MS in coating GaN platforms with plasmonic metals. Pulsed laser deposition (PLD) is an interesting and still not fully explored alternative for the fabrication of SERS substrates [37–38]. Hence, our studies reported herein aimed to investigate the influence of the method of Ag layer deposition on nanostructured GaN platforms on morphology, optical, and SERS enhancement properties of GaN/Ag SERS substrates. We first describe the fabrication processes using PLD and MS and discuss the influence of deposition process parameters on the morphology of fabricated Ag layers examined by scanning electron microscopy (SEM). Then, we present the results of their optical properties determined using UV–vis spectroscopy. Finally, we compare the SERS performance of the GaN/Ag substrates toward 4-mercaptobenzoic acid (pMBA) molecules adsorbed on them.

## Results and Discussion

### Fabrication of GaN/Ag substrates

**Figure 1:**
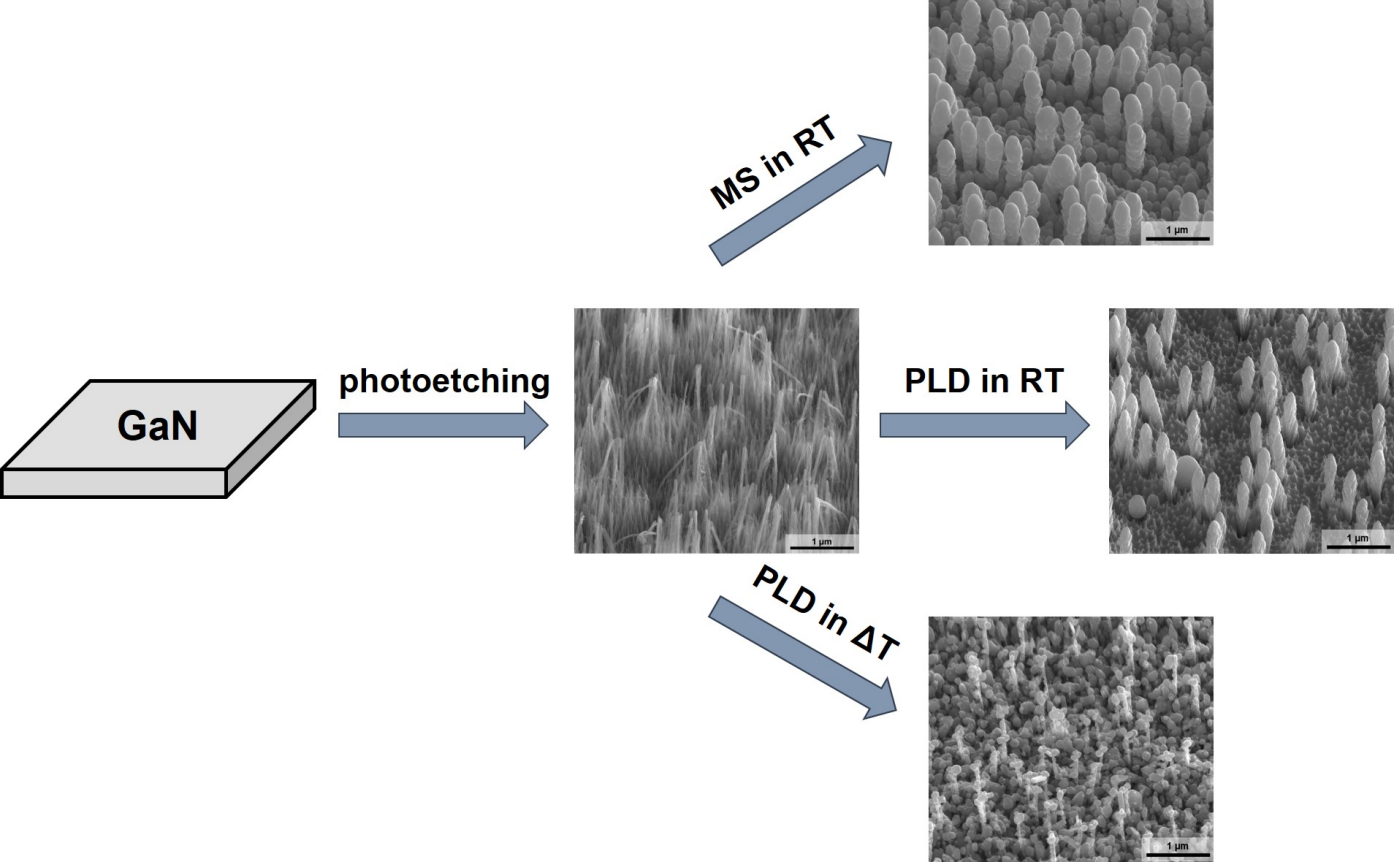
Overview of the fabrication process of GaN/Ag substrates.

GaN/Ag substrates were fabricated by Ag deposition on nanostructured GaN platforms using PLD and MS (Figure 1). In the first step, metal organic chemical vapor deposition (MOCVD)-grown GaN on sapphire epitaxial layers was exposed to photoetching following the procedure described elsewhere [31–32]. The surface morphology depends on the time of photoetching and the overall dislocation density [32]. Next, the fabricated nanostructured GaN platforms were coated with a Ag layer using PLD and MS to form GaN/Ag SERS substrates (Figure 1).

To compare SERS substrates fabricated using both methods, we attempted to prepare substrates with a comparable amount of deposited Ag, which was examined and controlled using atomic force microscopy (AFM). For this purpose, additional Ag layers were deposited on flat Si substrates. Based on the measured thickness of the Ag layers, calculations of the layer growth rate as a function of time for the MS method and the number of laser pulses for the PLD method were performed. In the next step, we calculated the number of laser pulses necessary to deposit by PLD Ag layers with a thickness comparable to Ag layers deposited using the MS method.

The thickness of Ag layers deposited on GaN platforms by MS was determined by the deposition time (Table 1). In the standard procedure developed in previous studies [28–36], a plasmonic metal layer is deposited for 280 s, forming a Ag layer with approximately 423 ± 5 nm thickness on a flat Si surface. In our studies, plasmonic metal was additionally deposited using one shorter and two longer deposition times to obtain GaN/Ag substrates with thinner and thicker Ag layers. As a result, GaN/Ag substrates were prepared with Ag layers deposited for 140, 280, 420, and 560 s, that is, with Ag layer thicknesses of 212 ± 2, 423 ± 5, 627 ± 4, and 833 ± 4 nm, respectively. The above thickness values were directly measured on a flat Si platform coated in the same processes as the GaN platforms. However, due to the different surface morphology of GaN platforms, the given thickness values correspond to the amount of deposited silver rather than the actual Ag layer thicknesses on GaN platforms. It is important to mention that when the Ag layer is too thin, the Raman peaks originating from the GaN platform may be visible in the SERS spectrum. From the point of view of SERS studies, the presence of peaks from the substrate in the spectrum is not desirable because it may hinder or prevent the correct interpretation of the spectra due to peak overlapping. Therefore, we did not study substrates with Ag layers thinner than 200 nm because the GaN material has an intense Raman signal.

**Table 1:** Parameters used during the deposition of the Ag layer by the MS method. The thicknesses of Ag layers were directly measured using AFM on flat Si platforms coated with Ag in the same processes as the GaN platforms.

Sample	Deposition time [s]	Temperature	Thickness of Ag layer [nm]

MS_1_RT	140	RT	212 ± 2
MS_2_RT	280	RT	423 ± 5
MS_3_RT	420	RT	627 ± 4
MS_4_RT	560	RT	833 ± 4

**Figure 2:**
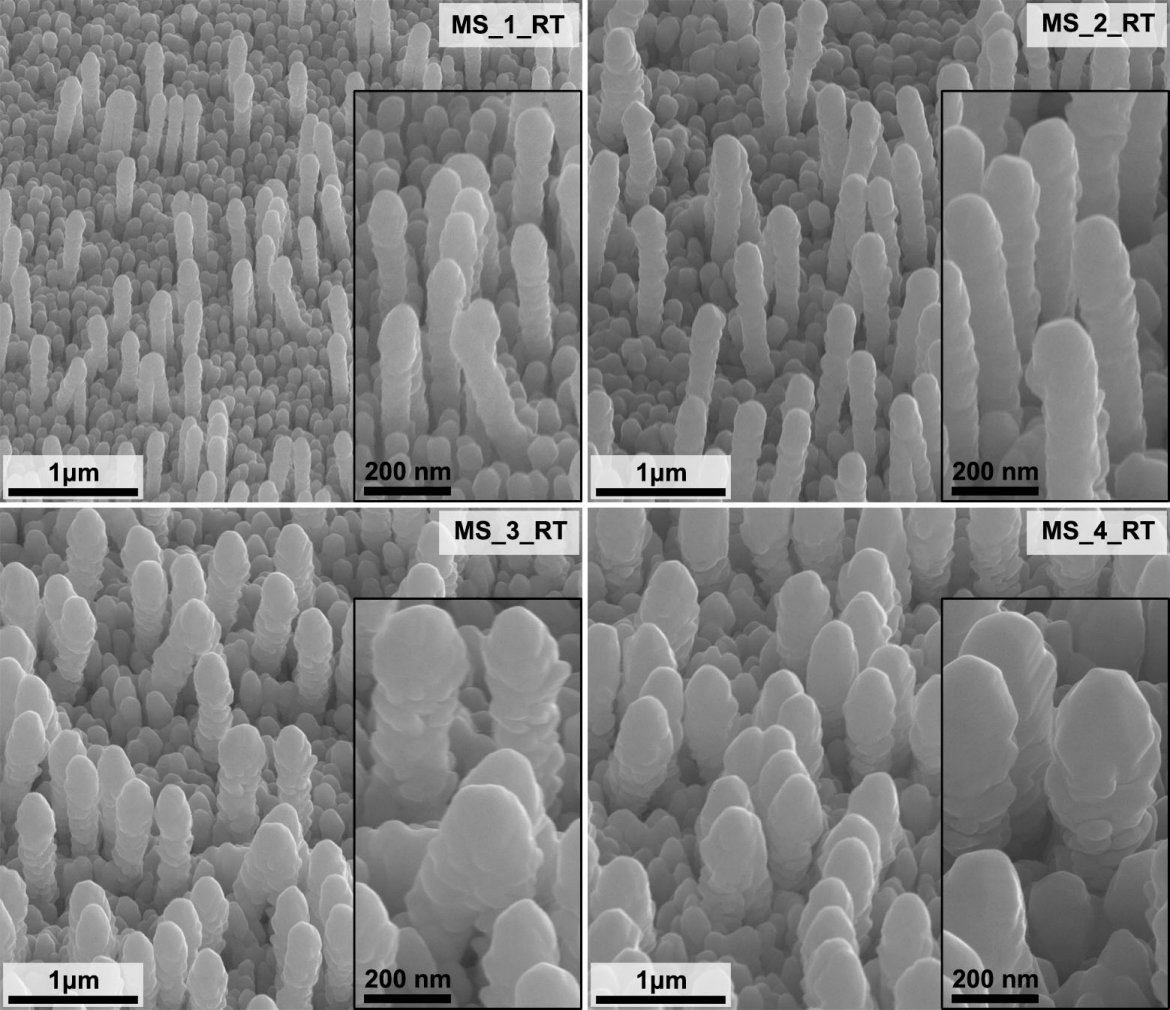
SEM images of the GaN/Ag substrates fabricated by deposition of Ag layers using the MS method at room temperature (RT) and different deposition times (Table 1). Images were taken at an angle of 45° to the plane of the sample. The inserts contain images with higher magnification.

SEM images of the GaN platforms with MS-deposited silver layers of different thicknesses are shown in Figure 2. The morphology of MS-fabricated SERS substrates does not change significantly with the change in Ag layer thickness. With a larger amount of deposited Ag, the thickness of the pillars increases, which translates into a reduction in the distance between them. The pillars are also becoming shorter because of the increasing metal layer thickness in the surrounding area.

**Figure 3:**
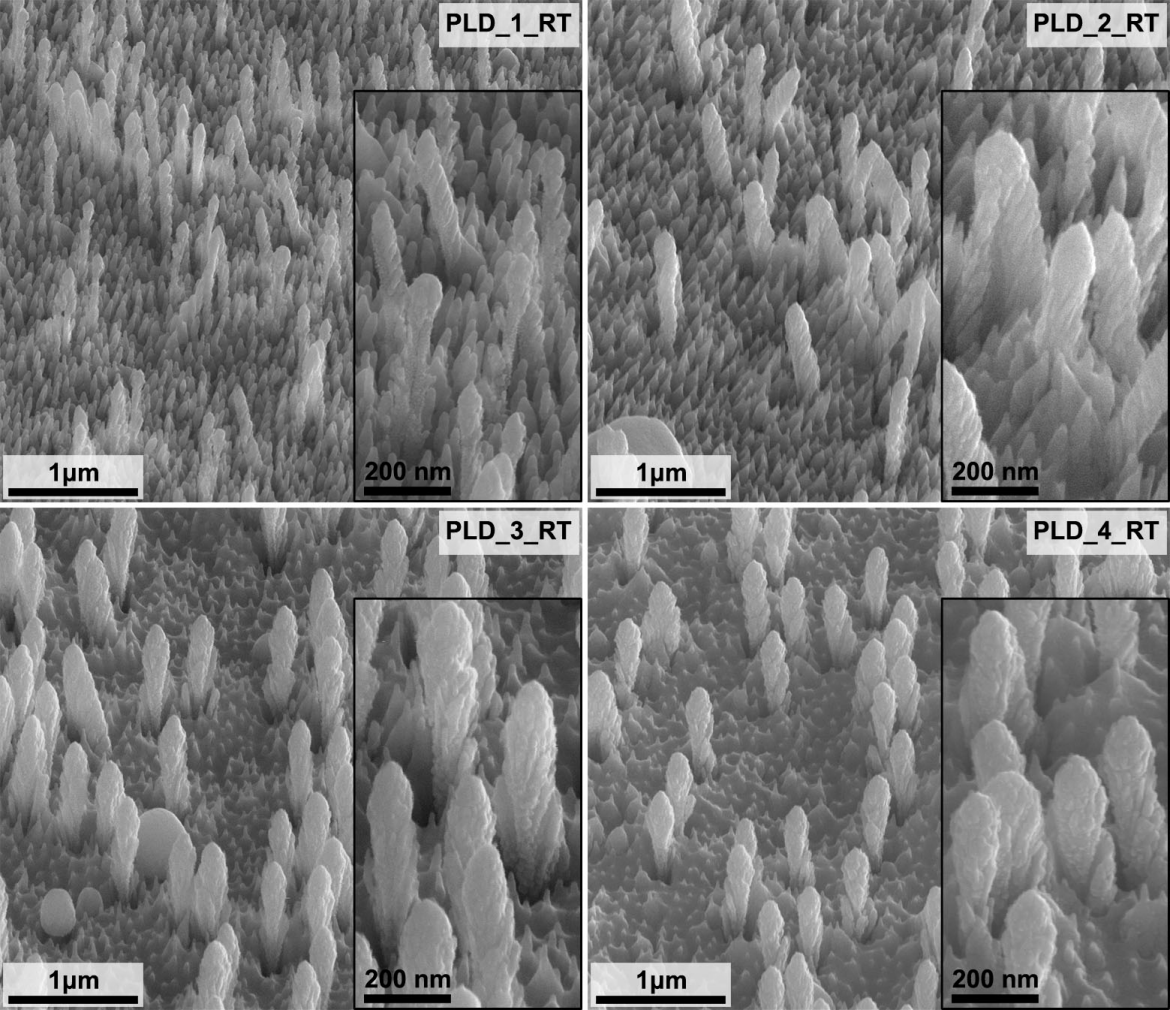
SEM images of the GaN/Ag substrates fabricated by PLD of Ag layers at room temperature with a different number of laser pulses (Table 2). Images were taken at an angle of 45° to the plane of the sample. The inserts contain images with higher magnification.

**Figure 4:**
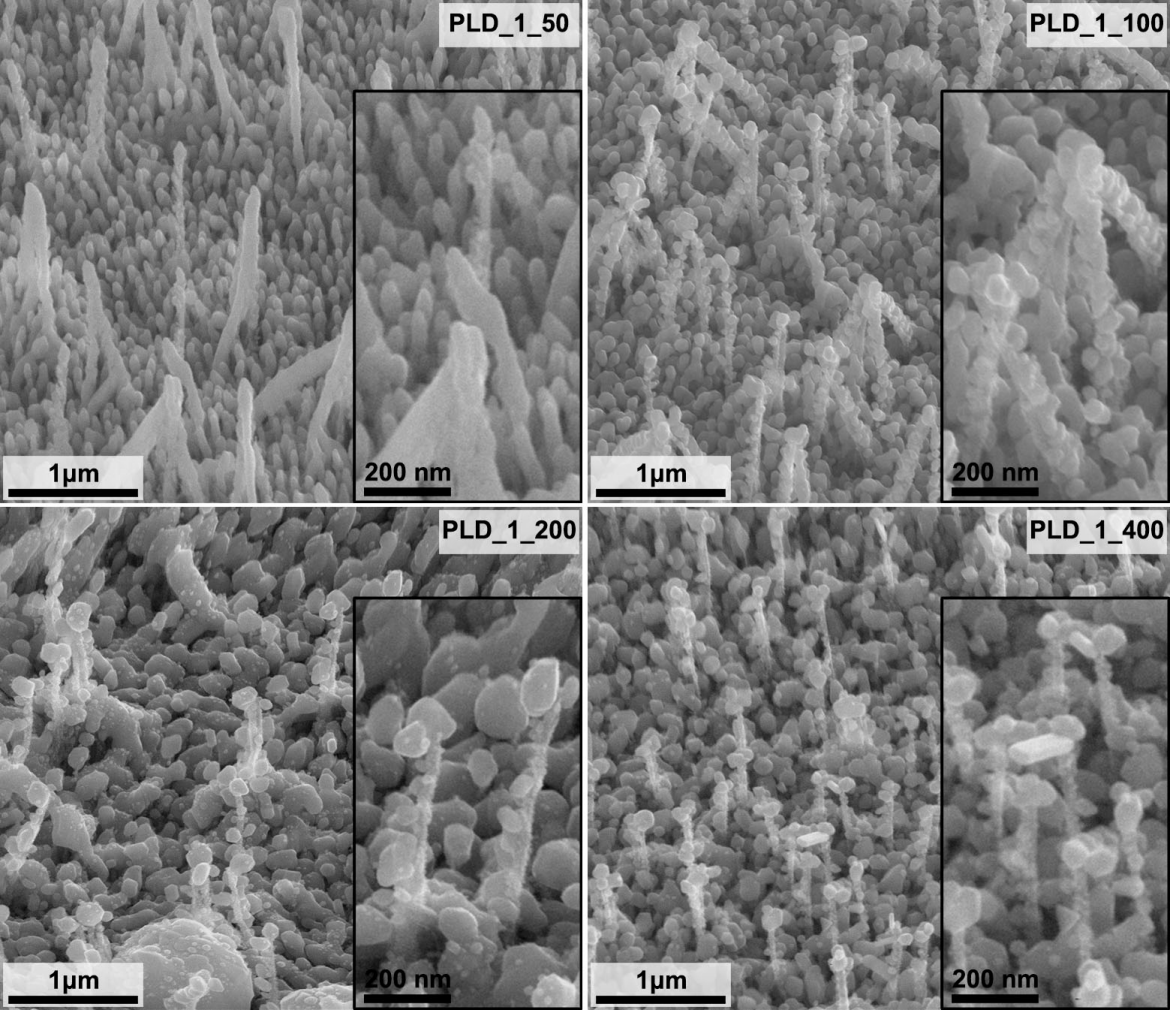
SEM images of the GaN/Ag substrates fabricated by deposition of Ag layers using the PLD method with 22000 laser pulses at different temperatures (Table 2). Images were taken at an angle of 45° to the plane of the sample. The inserts contain images with higher magnification.

Two sets of GaN/Ag SERS substrates were fabricated using the PLD method (Table 2, Figure 3 and Figure 4). Samples PLD_1_RT to PLD_4_RT were fabricated at room temperature using four different numbers of laser pulses, that is, 22000, 44000, 66000, and 88000. The Ag layer thicknesses estimated for this number of laser pulses are 215 ± 7, 429 ± 14, 644 ± 22, and 859 ± 29 nm, respectively. With this set of samples, we investigated the effect of the number of laser pulses on the morphology and the properties of obtained SERS substrates. In addition, for a selected number of laser pulses (22000), we deposited Ag layers on GaN platforms heated to four higher temperatures (50, 100, 200, and 400 °C), named samples PLD_1_50 to PLD_1_400. With this set of samples, we investigated how the temperature of the platform during the deposition process influences the morphology and properties of the SERS substrates. In the case of this set of samples, we used the same number of laser pulses for Ag deposition. Hence, we assumed the same Ag layer thickness of 215 ± 7 nm for all samples, equivalent to the thickness of the Ag layer deposited on a flat Si substrate at room temperature. The given thickness corresponds to the amount of deposited Ag determined by the number of laser pulses and does not reflect changes in the temperature of the platform. Determination of a numerical value describing the thickness of the Ag layer on nanostructured GaN platforms as a function of the temperature would be complicated because of the complex 3D morphology of the GaN platforms.

**Table 2:** Parameters used during the PLD of Ag layers. The thicknesses of Ag layers were estimated based on the calculated layer growth rate of 9.76 nm per 1000 laser pulses (linear approximation) on flat Si platforms.

Sample	Number of laser pulses	GaN platform temperature [°C]	Thickness of the Ag layer [nm]

PLD_1_RT	22000	RT	215 ± 7
PLD_2_RT	44000	RT	429 ± 14
PLD_3_RT	66000	RT	644 ± 22
PLD_4_RT	88000	RT	859 ± 29
PLD_1_50	22000	50 ± 2	215 ± 7
PLD_1_100	22000	100 ± 2	215 ± 7
PLD_1_200	22000	200 ± 2	215 ± 7
PLD_1_400	22000	400 ± 2	215 ± 7

SEM images of the GaN platforms with PLD-fabricated Ag layers of different thicknesses at room temperature are shown in Figure 3. The morphology of GaN/Ag substrates fabricated by PLD differs from that of substrates with comparable Ag layer thicknesses fabricated by the MS method. This is especially well pronounced on the surface surrounding the pillars. While for MS-fabricated GaN/Ag substrates, the area surrounding the pillars consists of rounded Ag structures, in the case of PLD-fabricated substrates, the pillars are surrounded by spiky Ag structures. Similarly, as for MS-fabricated substrates, with a larger amount of deposited Ag, the thickness of pillars increases, which translates into a reduction of the distance among them. They are also becoming shorter due to the increasing metal layer thickness in the surrounding area. The spiky structures surrounding the pillars are also becoming shorter and less pronounced. However, in contrast to the samples fabricated by MS, the surface of the Ag layer obtained by PLD is more developed and structured. As shown in Figure 3, pillars with a Ag layer have shapes similar to wheat ears. The Ag coatings formed on the pillars have preferred growth planes spatially oriented at a specific angle relative to the axis of the pillars. The observed effect can be associated with the initial orientation of GaN pillars to the plasma plume (Figure 1); pillars are under some angle to the plasma plume. This effect is similar to the one observed during the growth of thin metal films in oblique angle deposition [39]. However, in the case described herein, other parameters of the PLD process also influence the observed results. This way of growing the Ag layer on the pillars results in a thickness disproportion between the base and the top of the pillars, along with an increase in the amount of deposited Ag.

With the increasing amount of deposited Ag, the diameter of the pillars at the top can be about twice as large as at the base (samples PLD_3_RT and PLD_4_RT). This change in Ag pillar thickness occurs because the Ag layer, forming faster at the top of the pillars with the increase in the number of laser pulses, shadows the base of the pillars. It results in even faster growth of the Ag layer at the top due to the limited amount of Ag reaching the pillars’ base. The differences in Ag layer formation between MS and PLD can be explained by considering how the Ag plasma is formed. Plasma in MS is formed via Ag removal from the entire target surface, and the deposited material can move in different directions, not only perpendicular to the target surface. As a result, the sputtered Ag reaches the surface of the pillars from different directions and is deposited evenly on the pillars. In the PLD method, the target material is removed from a small area (in our case 2.03 ± 0.15 mm^2^) due to the impact of one laser pulse. A relatively narrow plasma stream is created, and most of the deposited material is directed perpendicularly to the coated substrate. The strongly directed stream of the deposited material seems to be the reason for the different morphology of the Ag layers deposited by PLD compared to MS.

SEM images of the GaN platforms with PLD-fabricated Ag layers of the same thickness but at different temperatures are shown in Figure 4. The morphology of the SERS substrates with a Ag layer deposited at 50 °C is similar to that of the SERS substrate deposited at RT (Figure 3 – sample PLD_1_RT). However, a further increase in GaN platform temperature during deposition significantly changes the morphology of the fabricated GaN/Ag substrates. Spiky Ag structures are not formed, and the metal layer on the pillars forms differently from the metal layer formed at RT.

First of all, with increasing GaN platform temperature Ag does not evenly cover all pillars and spiky structures on the surface of the GaN platforms. Deposited Ag forms structures resembling nanoparticles attached randomly to the platform’s surface. The dimensions of these nanoparticles increase with the temperature of the GaN substrate up to 200 °C, and, consequently, their number decreases. The largest Ag nanoparticles reach a diameter of 150–170 nm in the case of the PLD_1_200 sample. An increase in temperature also leads to an increase in the degree of crystallization of these Ag nanoparticles. At a temperature of 400 °C, more cubic and better crystallized nanoparticles are visible in the SEM images, but their dimensions are smaller than at 200 °C (Figure 4). The thermodynamic conditions of the Ag layer formation favor the crystallization and growth of Ag nanoparticles on the surface of the pillars and at the ends of the spiky structures surrounding them. The Ag nanoparticles formed at the ends of the spiky structures are located at small distances from each other or even coalesce. As a result, a porous, well-developed surface is created. Also, with the increase in the temperature of the GaN platforms, the surface of the pillars is less and less covered with Ag, and they look mostly exposed as a result of the growth of even larger Ag nanoparticles.

### Optical properties of fabricated GaN/Ag substrates

**Figure 5:**
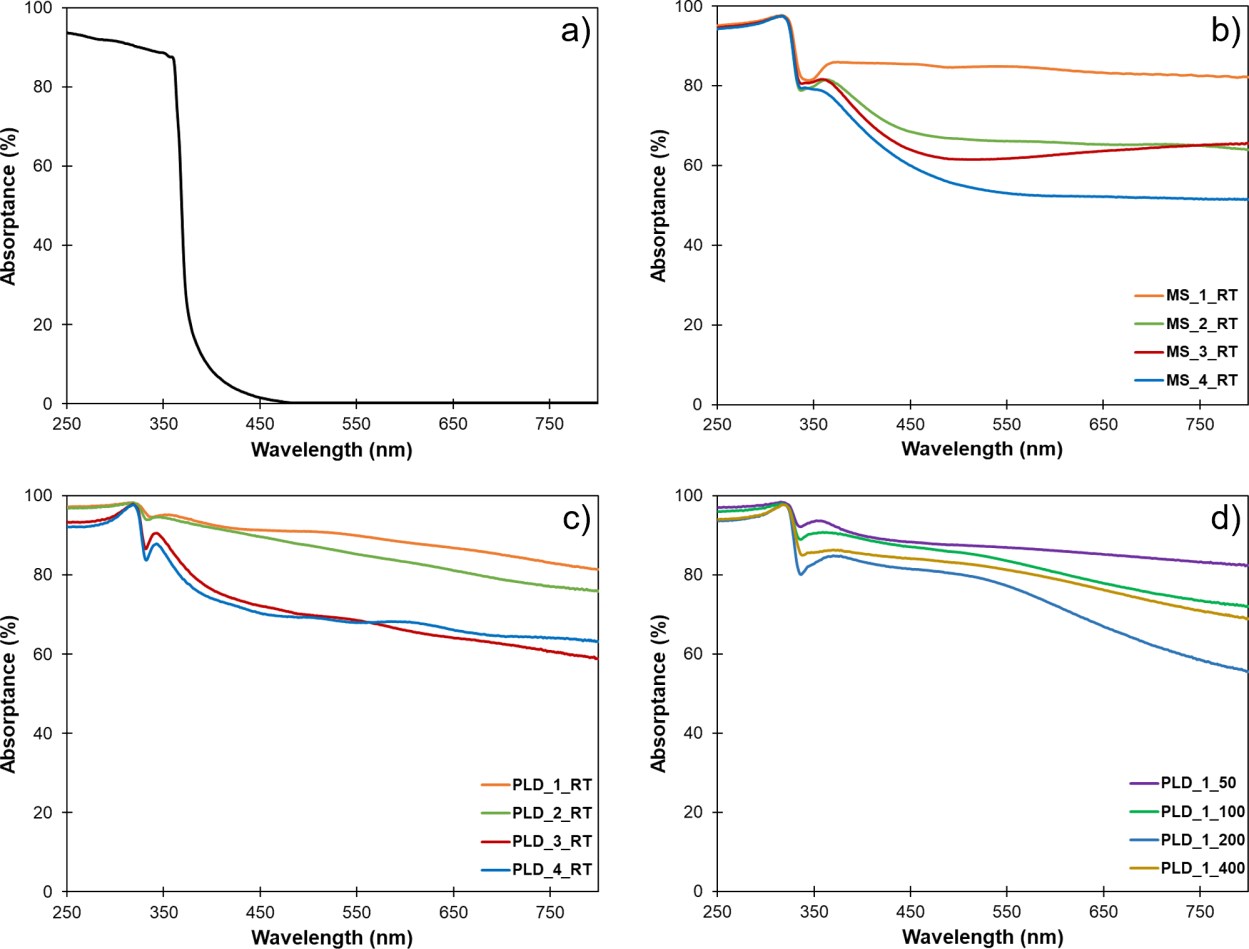
Absorptance spectra of the nanostructured GaN platform (a), GaN/Ag substrates fabricated using MS at room temperature and different deposition times (b), using PLD at room temperature with a different number of laser pulses (c), and using PLD with the same number of laser pulses at different temperatures (d).

The absorption spectra of the GaN platform and the GaN/Ag SERS substrates fabricated using MS and PLD at different temperatures are shown in Figure 5. The spectrum of GaN shows the typical spectral shape of a broadband semiconductor with an absorption edge around 365 nm (3.4 eV). The spectra of all GaN/Ag substrates show a strong absorption of light in the visible range, which is related to the phenomenon of surface plasmon resonance (SPR). The strongly nanoscale-rough surface causes a significant extension of the spectral range of SPR in comparison to thin silver layers. Based on the comparison of the light absorption level for samples in a given series, it can be seen that an increase in the amount of deposited metal causes a decrease in light absorption, which is related to a decrease in surface roughness and the filling of the nanoporous GaN structure with silver.

### SERS performance of fabricated of GaN/Ag substrates

**Figure 6:**
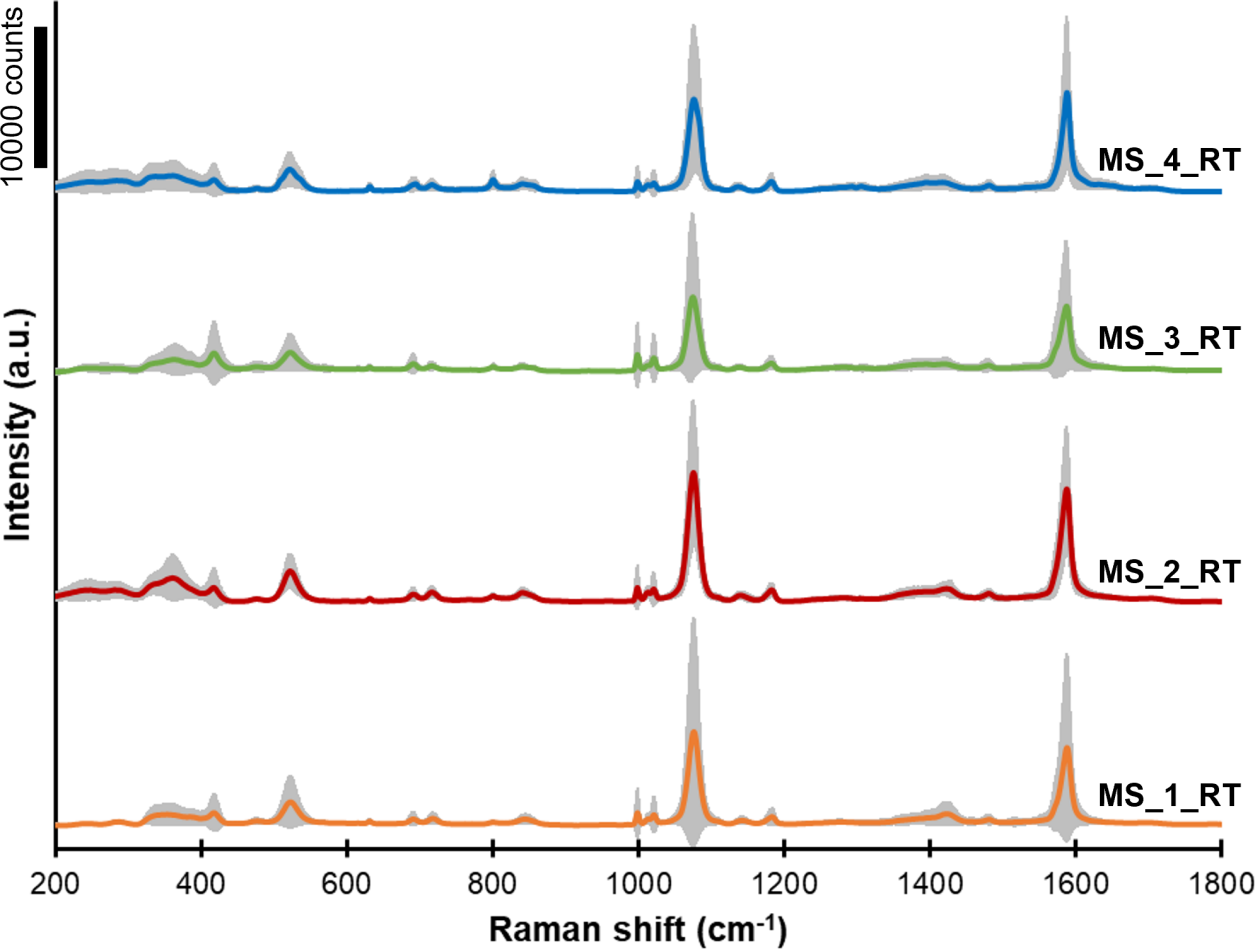
SERS spectra of pMBA adsorbed on GaN/Ag substrates fabricated using MS at room temperature and different deposition times (Table 1). The grey area in each spectrum represents the standard deviation of the signal.

**Figure 7:**
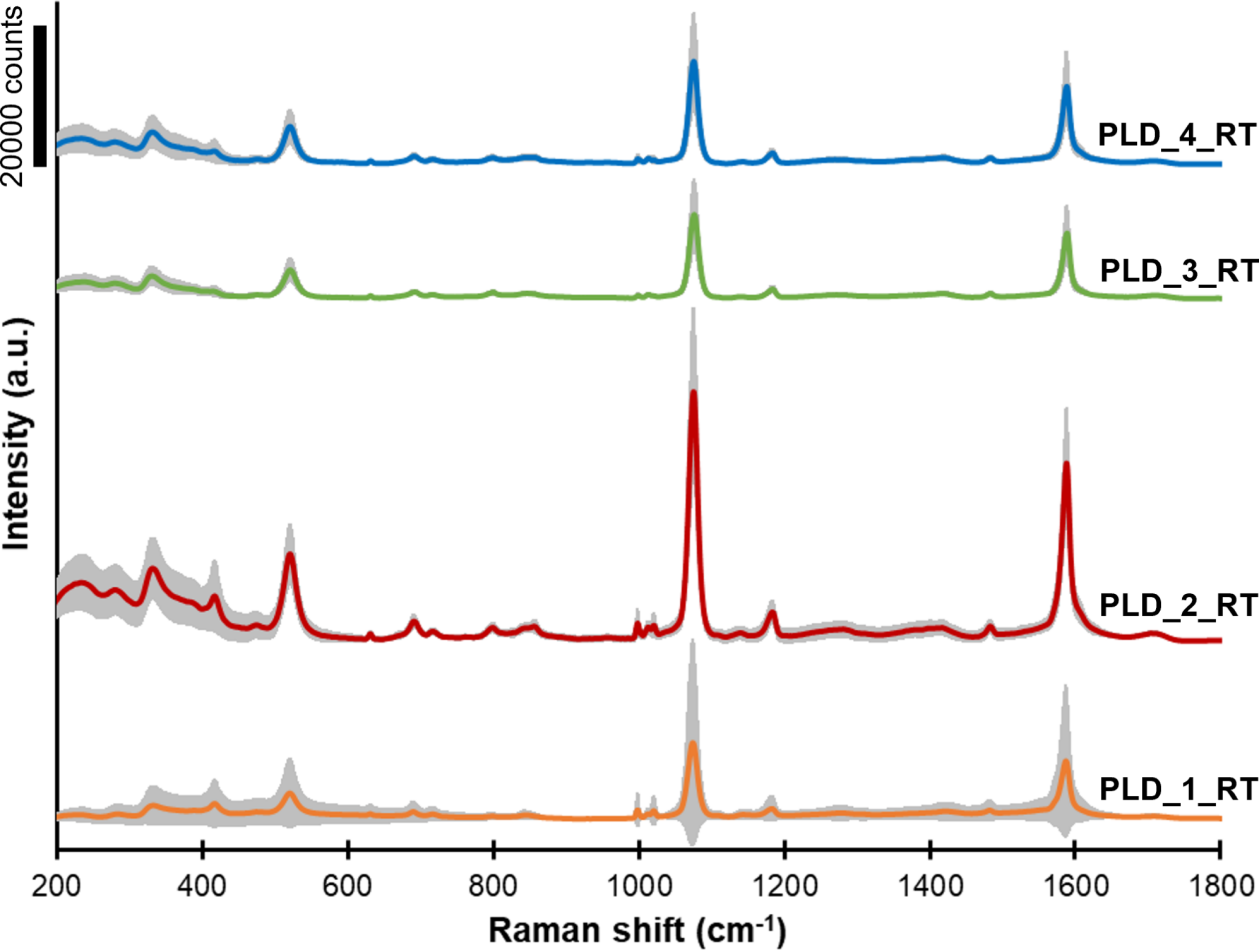
SERS spectra of pMBA on GaN/Ag substrates fabricated using PLD at room temperature with different number of laser pulses (Table 2). The grey area in each spectrum represents the standard deviation of the signal.

**Figure 8:**
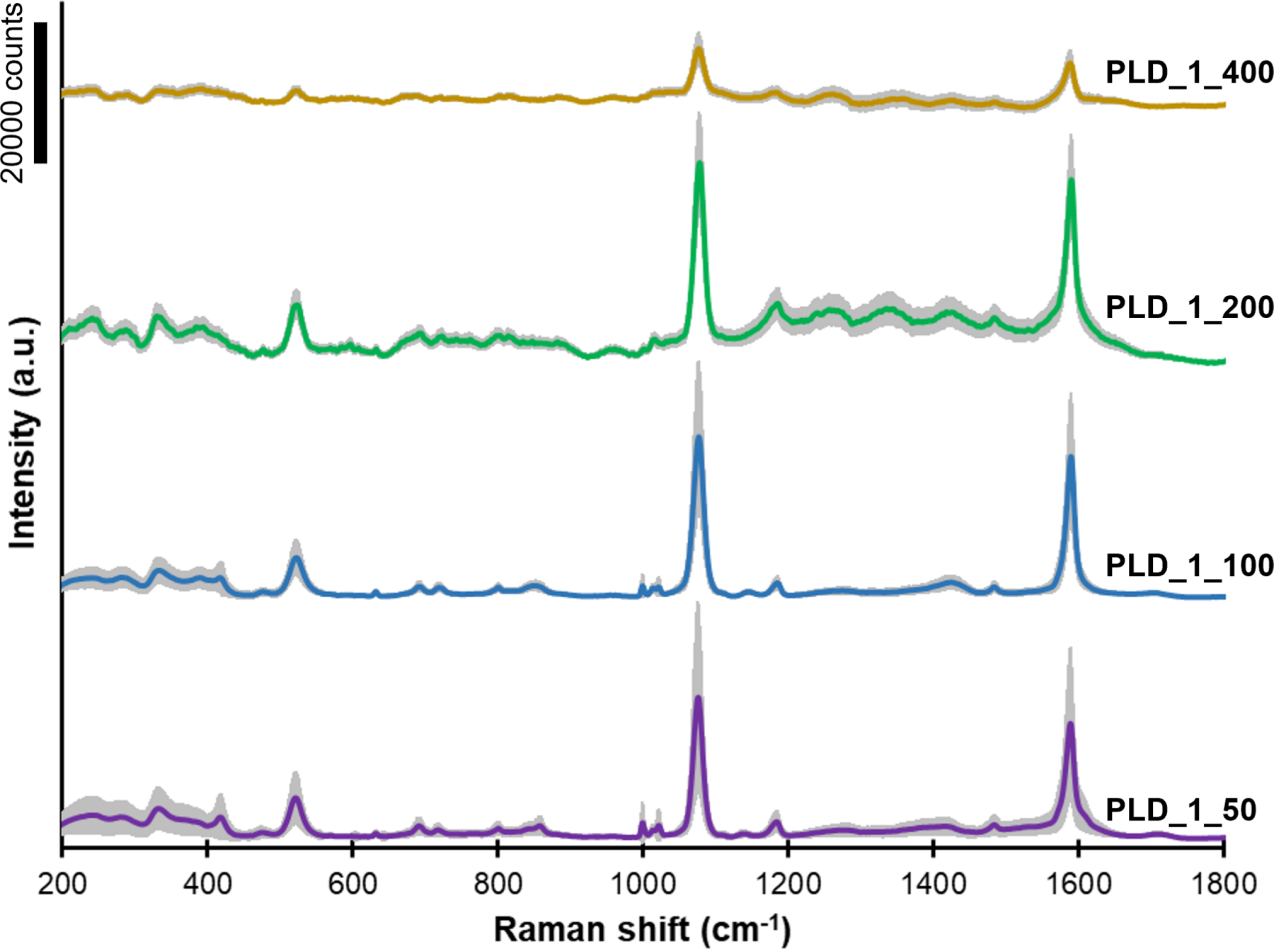
SERS spectra of pMBA adsorbed on GaN/Ag substrates fabricated using PLD with the same number of lasers pulses (22000) at different temperatures (Table 2). The grey area in each spectrum represents the standard deviation of the signal.

All fabricated GaN/Ag substrates were tested to assess their suitability for SERS applications using pMBA as a test analyte. Averaged pMBA SERS spectra acquired from GaN/Ag substrates fabricated using MS, PLD at room temperature, and PLD at higher temperatures are shown in Figure 6, Figure 7, and Figure 8, respectively. The colored lines represent the average spectra obtained for each SERS substrate, and the grey area represents the standard deviation of the average spectrum. Each spectrum was obtained by averaging the SERS spectra from at least 100 measurement points.

The evaluation of the average intensity of the peak at 1078 cm^−1^ and the standard deviation of intensity allowed us to determine which samples have the highest enhancement factor (EF) of the Raman signal and a high density of hot spots on the surface. The enhancement factor was calculated according to the formula described in [32]. Calculations were based on the intensity of the peak at 1078 cm^−1^, which is the most intense of all peaks in the pMBA spectrum. To determine EFs, a Raman measurement of pMBA adsorbed on the surface of a polished platinum plate was additionally made. The procedure for the analyte deposition on the SERS substrates and the platinum plate was the same [32]. The EF values calculated for fabricated SERS substrates are given in Table 3.

**Table 3:** Parameters of GaN/Ag substrates estimated based on SERS measurements.

Sample	Average intensity of the peak at 1078 cm^−1^ [arb. un.]	Intensity standard deviation of peak at 1078 cm^−1^	Enhancement factor (× 10^5^)

MS_1_RT	6742	8412	3.0
MS_2_RT	8495	5317	3.8
MS_3_RT	5334	6107	2.4
MS_4_RT	6808	5483	3.1
PLD_1_RT	10696	14674	4.8
PLD_2_RT	35344	11958	16.0
PLD_3_RT	11946	5138	5.4
PLD_4_RT	14610	6934	6.6
PLD_1_50	20126	13534	9.0
PLD_1_100	22856	10767	10.0
PLD_1_200	29236	7209	13.0
PLD_1_400	8470	2376	3.8

The SERS spectra of pMBA adsorbed on GaN/Ag substrates fabricated using MS are shown in Figure 6. The substrates fabricated by this method provide similar average peak intensities for pMBA, and no clear trend can be seen resulting from the deposition time used (Table 3). However, in some cases, the standard deviation of the 1078 cm^−1^ peak is equal to the average intensity. This observation suggests that at the microscale, SERS substrates produced by the MS method have regions characterized by very high and very low Raman signal enhancements. Therefore, these SERS substrates can be characterized by a lower density of hot spots on their surfaces than PLD-made substrates (Figure 7 and Figure 8). The EF calculated for the 1078 cm^−1^ peak has similar values in the range of (2.4–3.8) × 10^5^ for all samples fabricated using the MS method.

The pMBA SERS spectra acquired from GaN/Ag substrates fabricated using PLD at room temperature using a different number of laser pulses are shown in Figure 7. The intensity of the pMBA peaks for the PLD_1_RT, PLD_3_RT, and PLD_4_RT substrates is about twice as high compared to MS-fabricated substrates. The higher peak intensities are most likely due to the rougher metal surface formed and the spiky Ag structures on the surface of the GaN/Ag substrates. In the case of Ag deposition by MS, the thermodynamic conditions favored the growth of oval Ag structures on the GaN platform. The rounded and smooth Ag structures gave lower enhancement to the Raman signal. Therefore, the PLD method allows for the fabrication of GaN/Ag substrates with better enhancement properties. The EF for samples PLD_1_RT, PLD_3_RT, and PLD_4_RT increases significantly from 4.8 × 10^5^ for PLD_1_RT to 6.6 × 10^5^ for PLD_4_RT. The PLD_2_RT substrate is characterized by the highest EF (16.0 × 10^5^) in the entire series of the discussed substrates, which may result from Raman scattering enhancement on the pillars and spiky structures on the surface of the GaN/Ag substrates. In the case of the PLD_2_RT sample, the spiky structures on the surface are very numerous, tall, and sharply pointed. On the surface of the PLD_1_RT sample, they are equally numerous but not as sharply pointed. As the amount of deposited Ag increases (samples PLD_3_RT and PLD_4_RT), these structures are covered by a thicker layer of Ag and become lower and less visible. As a result, their contribution to the Raman scattering signal enhancement is smaller. A comparison of signal standard deviations of the SERS spectra obtained for PLD-fabricated substrates made at room temperature indicates that substrate PLD_1_RT has a much lower density of hot spots than the other substrates in the series (Figure 7).

GaN/Ag substrates fabricated by the PLD method at higher GaN platform temperatures using 22000 pulses were also tested (Figure 8). The temperature of the GaN platform during the deposition of Ag layers has a significant impact on the morphology of Ag layers and, thus, on the calculated EF. Comparing these results with the SEM images (Figure 4) reveals the correlation between the morphology of the formed Ag layers and the EF. The EF increases with increasing temperature and was calculated to be 9.0 × 10^5^, 10.0 × 10^5^, and 13.0 × 10^5^ for the samples prepared at 50, 100, and 200 °C, respectively. As we wrote earlier, an increase in temperature favors the formation of larger Ag nanoparticles. Probably because of this, the distances among them are smaller, and more hot spots are created, which provide a greater enhancement of the Raman signal, consistent with the theory presented in numerous publications [40–41]. The SERS substrate made at 400 °C deviates from the visible trend as its EF is only 3.8 × 10^5^. This observation correlates the most with the predicted highest degree of crystallization of the formed Ag nanoparticles because the sample is not distinguished in terms of the size and distribution of Ag nanoparticles.

An advantage of the GaN/Ag SERS substrates fabricated by PLD over those fabricated by MS is a much lower standard deviation of the intensity of the measured peaks in the spectra presented in Figure 7 and Figure 8. The exceptions are samples PLD_1_RT and PLD_1_50 covered with the same amount of Ag at room temperature and 50 °C, characterized by a very similar morphology of the Ag layer.

**Figure 9:**
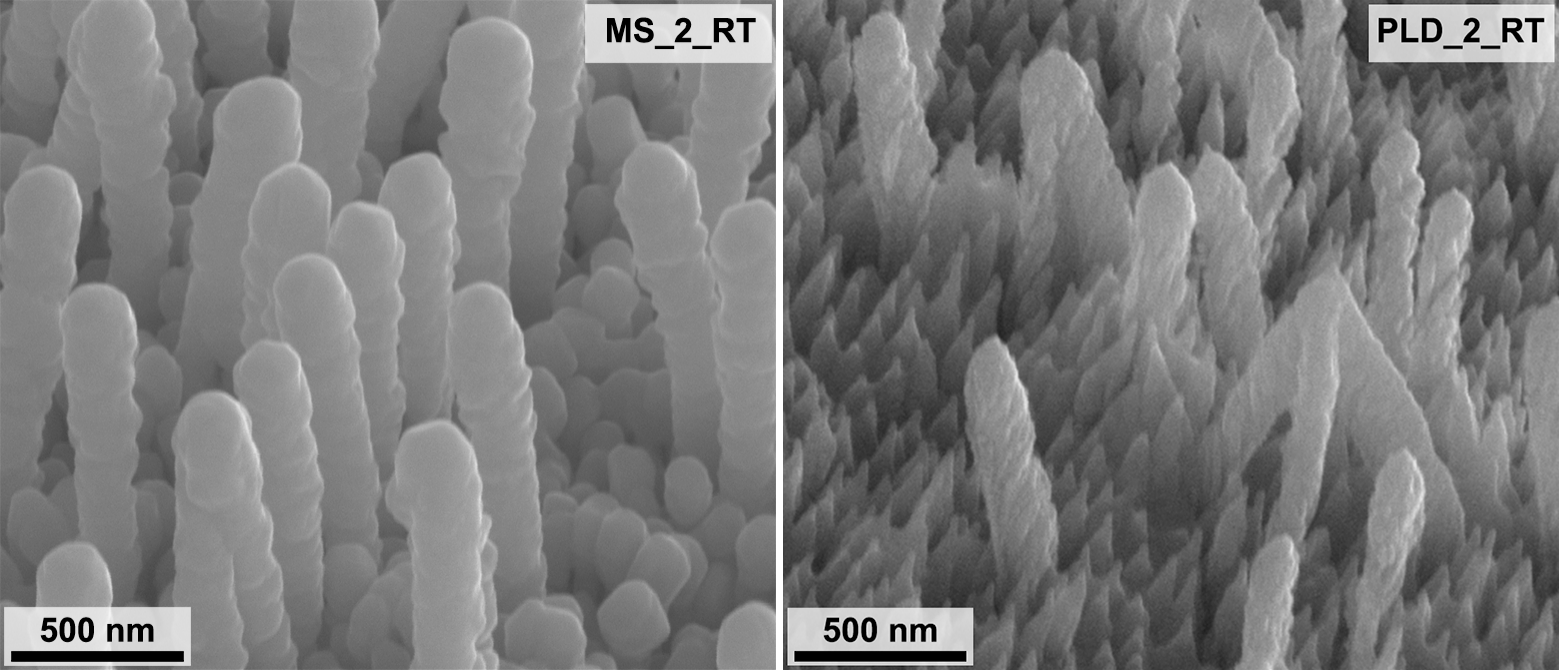
SEM images of GaN/Ag substrates, MS_2_RT (left) and PLD_2_RT (right), with a comparable thickness of the reference Ag layer (423 ± 5 nm and 429 ± 14 nm, respectively) and with the highest enhancement factor in the group of samples prepared by MS and PLD. Images were taken at an angle of 45° to the plane of the sample.

In summary, using the PLD method, we fabricated a GaN/Ag substrate with about 4.4 times higher EF than the best MS-fabricated substrate (Table 3). As seen on the high-magnification SEM images of silver layers formed by MS and PLD coating of GaN nanopillars, there is a big difference in the morphology of Ag layers (Figure 9). The MS layer is almost continuous, though rounded metal structures can still be discerned. In contrast, the PLD coating formed metal structures with a more complex morphology that shows sharp edges, spikes, and overall porosity. Such diversified morphology of plasmonic metals (shape and distance between particles) is known to introduce more hot spots on substrates used for SERS [42–44]. Therefore, it can be the reason for one order of magnitude higher EFs on SERS substrates fabricated using PLD. Another critical issue is the increase of the EF at a lower amount of Ag deposited by PLD compared to the MS method. The sample PLD_1_RT, made with half as much Ag as the MS-made sample MS_2_RT, with the highest EF among other MS-made samples, has an EF that is 1.3 times higher. However, a more significant EF increase is observed for SERS substrates made by PLD deposition on heated GaN platforms. The PLD_1_200 sample has an EF 4.4 times higher than that of MS_2_RT despite reducing the amount of deposited Ag by half.

## Conclusion

In this work, we investigated how the fabrication method of GaN/Ag SERS substrates, that is, pulsed laser deposition or magnetron sputtering, influences their morphology and, as a result, their optical and SERS properties. In the case of the PLD-fabricated SERS substrates, we also investigated the effect of heating the GaN platform during deposition. Ag layers of comparable thicknesses deposited on nanostructured GaN platforms using PLD and MS had different morphologies, particularly evident in the area surrounding the pillars. While for MS-fabricated substrates, the area surrounding the pillars consisted of rounded Ag structures, in the case of PLD-fabricated substrates, pillars are surrounded by spiky Ag structures. Heating of the GaN platform during PLD fabrication of Ag layers significantly influences the morphology of the GaN/Ag substrates, especially at temperatures of 100 °C and above. Instead of spiky Ag nanostructures, nanostructures with irregular, but more rounded, shapes are obtained. The optical properties of the GaN/Ag substrates, determined by UV–vis spectroscopy, are affected by their morphology. All fabricated substrates absorb light in the whole UV–vis region, to different extents ranging between 60% and 90%. SERS properties of the GaN/Ag substrates were evaluated by measuring SERS spectra of 4-mercaptobenzoic acid (pMBA) molecules adsorbed on them. Comparison of GaN/Ag substrates with comparable thicknesses of the Ag layers showed that for all SERS substrates obtained by the PLD method, the enhancement factors were higher than for SERS substrates obtained by the MS method. All estimated EFs ranged from 2.4 × 10^5^ to 3.8 × 10^5^ for MS-fabricated substrates and from 3.8 × 10^5^ to 16 × 10^5^ for PLD-fabricated substrates. The highest EF obtained for the PLD-fabricated GaN/Ag substrates (sample PLD_2_RT) was over four times higher than that for the MS-fabricated GaN/Ag substrates (sample MS_2_RT). The better SERS properties of substrates with Ag layers deposited by PLD were attributed to their markedly different morphology, especially to the sharp edges and spikes.

## Materials and Methods

### Fabrication of GaN/Ag substrates

Two physical vapor deposition methods, MS and PLD, were used to coat GaN platforms with Ag layers. The research aimed to compare the enhancement of the Raman signal on GaN/Ag SERS substrates fabricated using MS and PLD under various conditions. The nanostructured GaN platforms and GaN platforms with MS-deposited Ag layers were fabricated at the Institute of High-Pressure Physics of the Polish Academy of Sciences following a procedure described previously [30–31]. In the first step, nanostructured GaN platforms were prepared from commercial MOCVD-grown GaN on sapphire wafers by photoetching [45]. The 5 μm thick GaN layers were n-type with a carrier concentration *n* = 1 × 10^18^ cm^−3^ and a dislocation density of about 8 × 10^8^ cm^−2^. The 5 × 5 mm samples were cut from 3″ MOCVD-grown GaN on sapphire wafers. The cut samples were cleaned in organic solvents and rinsed in de-ionized water. Samples were then photoetched in stirred KSO-D solution (0.02 M K_2_S_2_O_8_ + 0.02 M KOH aqueous solution) under illumination of a 300 W UV-enhanced Xe lamp (Oriel, Newport, UK). This photoetching procedure results in the formation of nanocolumns on line defects (dislocations), as was demonstrated in [45]. The nanostructured GaN platforms were then coated with Ag using a Quorum Q150TS sputter coater (Quorum Technologies Ltd., Laughton, UK) with a cleaning oxidized target function engaged [32]. The thickness of the Ag layers was changed by using the deposition time in the range of 140–560 s. As a result, four samples with different Ag layer thicknesses were made, corresponding to deposition times of 140, 280, 420, and 560 s.

The PLD coating of the GaN platform with silver layers was performed at the Institute of Optoelectronics of the Military University of Technology. For preparing the GaN/Ag substrates, a PLD system with an ArF excimer laser (LPX Pro 305, Lambda Physik AG, Göttingen, Germany) was used. The following parameters characterize the laser used in the experiment: λ = 193 nm, *E* = 600 mJ, and τ =15–25 ns. The deposition was carried out in a vacuum chamber equipped with an oil-free pumping system enabling a pressure of 10^−5^ mbar. Constant pressure conditions (4.60 ± 0.65 × 10^−5^ mbar), the same laser repetition rate (5 Hz), and a constant energy of the laser pulses (265 ± 3 mJ) were used in all experiments. The laser radiation fluence on the target surface, calculated based on the laser focus area (2.03 ± 0.15 mm^2^) and the laser pulse energy, was 14.03 ± 0.94 J/cm^2^. The value of the laser radiation fluence determines the mass of silver deposited on the substrate and, therefore, the growth rate of the Ag layer per laser pulse, affecting the size and shape of the formed silver nanostructures. Only the number of laser pulses was changed to obtain Ag layers of different thicknesses, and the temperature of the substrates was varied to change the morphology of the deposited Ag layers (RT, 50, 100, 200, and 400 °C) (Table 2). In these studies, four GaN/Ag substrates with different thicknesses of the Ag layer (corresponding to 22000, 44000, 66000, and 88000 laser pulses) deposited at room temperature, and four substrates with the same amount of deposited Ag (corresponding to 22000 laser pulses) but at different GaN platform temperatures (50, 100, 200, and 400 °C) have been performed. The number of laser pulses in subsequent experiments was calculated in such a way that the thicknesses of the Ag layers were comparable with the thicknesses obtained for samples prepared by the MS method. A rotating silver target was used for metal layer deposition with a purity of 99.95% (Mint of Poland, Warsaw, Poland). The laser beam was focused on the target at an incident angle of 45°, and the distance between the target and the GaN platform was constant and equal to 65.0 ± 0.5 mm.

### Characterization of GaN/Ag substrates

The morphology of the fabricated GaN/Ag substrates was visualized using a scanning electron microscope (SEM) (Quanta 3D FEG, FEI Company, Eindhoven, Netherlands). The thickness of the reference Ag layers, deposited on flat silicon substrates at room temperature, was measured with an atomic force microscope (AFM) (NT-MDT, Moscow, Russia) in non-contact mode using the approach described previously [37]. The silver layers were removed randomly on the sample to form a sharp edge for measurement of height (layer thickness). AFM measurements were carried out in three different areas on the surface of each sample. Ten AFM cross sections from different scanning areas were made and averaged for each sample, from which the average layer thickness and the standard deviation of thickness were determined. To measure the thickness of Ag layers deposited by MS, Ag was deposited in the same process on GaN platforms and flat Si substrates. In the case of PLD, based on the results of the Ag layer thickness measurement on additional Si reference samples prepared in the first stage of the research, the Ag layer growth rate per 1000 pulses was calculated. Using the growth rate obtained this way, we calculated the number of laser pulses necessary to obtain Ag layers with thicknesses comparable to those fabricated using the MS method. It must be mentioned that the thickness of the Ag layer on the vertically oriented GaN pillars is markedly smaller than that of the Ag layer on the surrounding surface. From SEM images, it could be estimated to be almost one order of magnitude smaller.

The optical properties of GaN/Ag substrates were investigated using a Lambda 650 UV–vis spectrophotometer (Perkin Elmer, Waltham, MA, USA) equipped with a 150 mm integrating sphere. The samples were measured at room temperature in the 250–800 nm spectral range with an increment of 1 nm. The transmittance (%T) and the reflectance (%R) of the GaN/Ag substrates were measured. The absorptance (%A), defined as the fraction of incoming radiant flux absorbed by an object, was calculated via %A = 100% − %T − %R.

### SERS measurements

The SERS spectra were acquired using a Renishaw inVia Reflex Raman microscope (Renishaw Plc, Wotton-Under-Edge, UK) equipped with an EMCCD detector (Andor Technology Ltd, Oxford Instruments, Belfast, UK). The measurements were carried out using laser radiation with a wavelength of 785 nm and a 50× objective lens (N.A. = 0.75). The spectrometer was calibrated using an internal silicon wafer, and the spectrum was centered at 520.5 cm^−1^. The measurement parameters were as follows: acquisition time of 1 s and one accumulation at each point for GaN/Ag substrates, and acquisition time of 5 s and 50 accumulations at each point for a polished platinum plate. The SERS spectra were acquired from at least 100 points. All collected spectra were processed in the WiRE 5.5 software and then averaged using the CasaXPS software. The SERS measurements were made on SERS substrates that were first embedded in 0.001 M solution of 4-mercaptobenzoic acid (pMBA, Sigma Aldrich, St. Louis, MO, USA) in ethanol, then thoroughly washed with ethanol and dried in the air. The detailed procedure of applying the analyte on the SERS substrate was described elsewhere [37]. The enhancement factors (EFs) for all GaN/Ag substrates were calculated using the equation described elsewhere [32].
